# Immunometabolism, an emerging field in perioperative and critical care medicine: a narrative review

**DOI:** 10.1016/j.bja.2026.01.003

**Published:** 2026-02-03

**Authors:** Isabell Nessel, Victoria S.K. Tsang, Johannes Schroth, Henrike Janssen

**Affiliations:** Faculty of Medicine and Dentistry, William Harvey Research Institute, Queen Mary University of London, London, UK

**Keywords:** critical care, immunometabolism, immunonutrition, leucocyte, perioperative stress

## Abstract

Metabolism and immune function are tightly intertwined, and have been the focus of research in the expanding field of immunometabolism. Immunometabolism focuses on the metabolic adaptation of immune cells to their environment, allowing an efficient and targeted immune response even in hypoxic or nutrient-depleted tissue. The inflammatory response can be pathologically altered in chronic metabolic disease or acute injury as a result of poor immunometabolic adaptation, contributing to short- and long-term complications. Novel techniques, including ‘omics’ investigations, advanced imaging, and phenotyping with flow cytometry, now allow for in-depth and near real-time profiling of the intricate interactions between cell function and metabolism. Although this has significantly increased our knowledge of immunometabolic consequences in the fields of diabetes mellitus and cancer, it has been largely underappreciated in the perioperative period, even though the perioperative period serves as a strong translational model to investigate complex and highly dynamic metabolic shifts in acute inflammation. Interventions to modulate immunometabolism are being explored, particularly through immunonutrition in critical care, with heterogeneous results underscoring the need for greater understanding of the complex underlying mechanisms. In this review, we describe immunometabolic adaptation in health and metabolic disease, and under the acute inflammatory stress caused by surgery. We also discuss potential interventions, including immunonutrition and mode of anaesthesia to modulate immunometabolism in the perioperative period and critical illness.

**Editor’s key points**

• Immunometabolism focuses on the metabolic adaptation of immune cells to their environment.
• In acute infammatory states such as surgical injury, and chronic inflammatory states such as diabetes mellitus, this metabolic adaption may be altered.
• Interventions to modulate immunometabolism, including through anaesthetic choices and immunonutrition, are being explored.
• Further research is required into the basic and translational science of immunometabolism.

Immunometabolism explores the bidirectional influence of metabolism and immune cell functionality. Besides simply providing energy in form of adenosine triphosphate (ATP) to cells, metabolic pathways and products play an important role in immune cell adaptation as part of the inflammatory response. Many recent findings of this relationship are the result of in-depth analysis using novel techniques, such as metabolomics, imaging, and flow cytometry (Box 1).^1^^,^^3^^,^^7^

Box 1 **Techniques to investigate immune cell metabolism.**

*Flow cytometry-based assays:* The flow cytometry methods SCENITH and CENCAT rely on quantification of cellular translation,^1^^,^^2^ whereas others characterise metabolic state by the expression of key metabolite transporters and rate-limiting enzymes.^3^^,^^4^ Metabolite uptake can be measured using biorthogonal chemistry, such as glutamine uptake by the SLC1A5 transporter.^5^ Dyes can also be used to interrogate mitochondrial function and lipid droplets.^6^

*Metabolic flux and functional assays*: The Seahorse (Agilent) metabolic analyser measures real-time oxygen consumption and pH levels to infer metabolic activity in bulk samples of cells.^7^ Cellular metabolic flux can be investigated using stable isotope tracing of glucose, glutamine, palmitate, pyruvate, or arginine.^8^^,^^9^

*Genomic and transcriptomic approaches:* Functional genomics methods such as CRISPR-screens allow mechanistic interrogation of cellular metabolism,^10^ whereas scRNA-seq based methods have been used to analyse metabolic effector programmes in leucocytes.^11^

Alt-text: Box 1

Metabolism and the inflammatory response are closely intertwined and dynamic in nature, continuously adapting to external and internal cues of injury. However, chronic, acute, or highly localised metabolic and inflammatory stress may also result in dysregulation of this relationship. Chronic systemic metabolic disease, such as obesity and type 2 diabetes mellitus, fuels low-grade inflammation contributing to associated complications.^12^^,^^13^ Acutely disturbed metabolism, such as hyperglycaemia, during and after surgical injury, and in critical illness, is common and adversely affects outcomes.^14^^,^^15^ Cancer cells and the tumour immune microenvironment are highly adaptable to local metabolic influences, driving cell reprogramming with major consequences for tumour growth and metastasis.^16^^,^^17^ With a rising incidence of metabolic disease in the general population,^18^ more patients will face acute inflammatory insults on top of underlying low-grade chronic inflammation with metabolic maladaptation and higher susceptibility to complications.^19^^,^^20^

Findings from studies conducted in sepsis highlight the complex immunometabolic reprogramming of immune cells in host defence.^21^ Adaptations in sepsis encompass overarching illness behaviour, organ dysfunction, and cellular reprogramming, all set within a catabolic metabolic state.^21^ Many of these aspects are shared by other forms of critical illness and acute inflammatory insults, such as surgery. However, immunometabolism remains under-investigated in the perioperative period. This is despite the fact that elective surgery represents one of the best translational models of acute inflammation, as it allows for serial investigations before and after surgical inflammatory stimuli.^22^ Applying novel techniques^1^^,^^3^^,^^7^ to investigate immunometabolism in clinical scenarios relevant to perioperative medicine is therefore not only expanding our knowledge of the interaction of metabolism and inflammation in acute inflammatory stress, but may also identify new therapeutic strategies.

This review leverages the extensive mechanistic data from critical illness to justify and guide future research into the unique environment of the perioperative period. We lay out in detail the fundamental physiological pathways of immunometabolism at steady-state and upon activation. We describe major immunometabolic consequences in obesity and diabetes mellitus, discuss immunometabolism, and, where possible, highlight potential interventions in the perioperative period and in critical illness. Throughout, we update readers on current clinical trials investigating immunometabolic interventions.

## Immunometabolism and its regulation

### Immunometabolism and core metabolic pathways

Immunometabolism refers to metabolic adaptations of immune cells in the physiological immune response and in pathological immune dysregulation.^23^ In the non-activated state, immune cells maintain cell integrity through a combination of metabolic pathways specific to the cell type. Upon activation, metabolism is reprogrammed to meet new, increased demands for necessary functions such as cytokine production or proliferation.^24^ This flexibility of switching metabolic pathways, initiated by environmental cues, master regulators, and metabolic byproducts, is termed ‘metabolic plasticity’ and is described in more detail in this review.^23^

**Fig 1:**
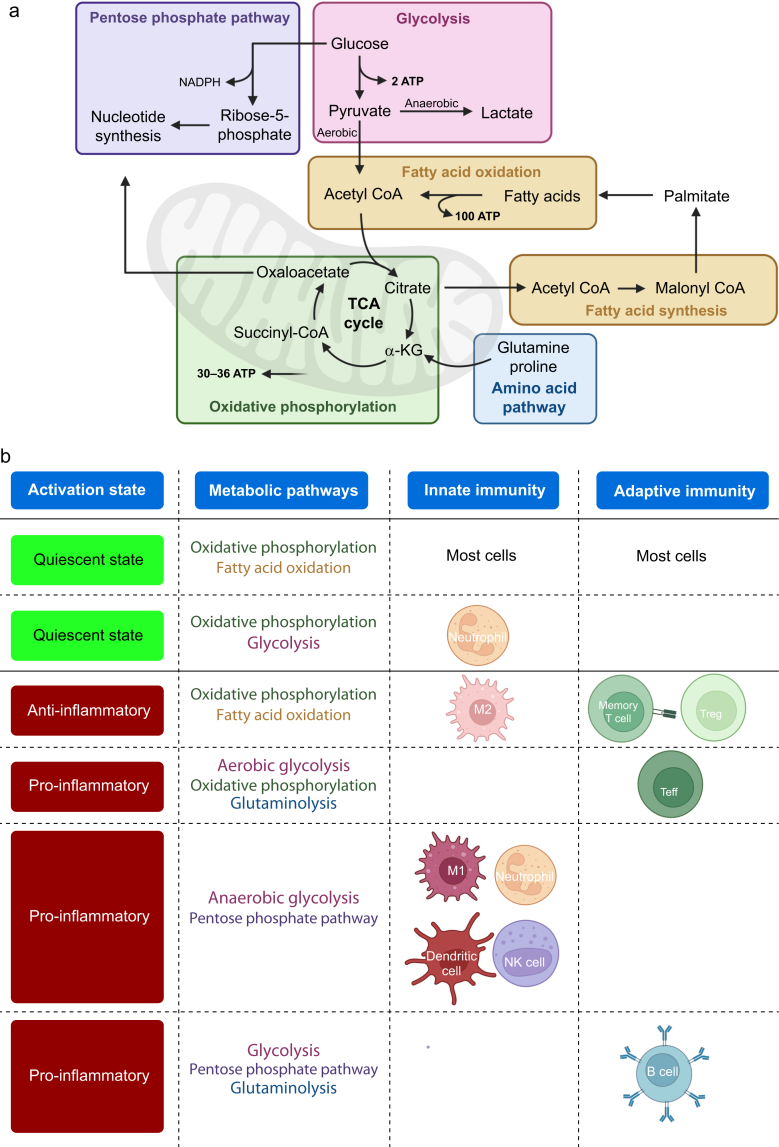
Metabolic pathways and their associations with immune cell activation. (a) Schematic overview of key substrates and their utilisation in major metabolic pathways that generate energy (adenosine triphosphate [ATP]), reducing equivalents and biosynthetic intermediates, essential for cellular functions and growth. (b) Predominant metabolic pathways used by innate and adaptive immune cells during homeostasis, and their metabolic reprogramming in the perioperative period or during critical illness. CoA, coenzyme A; NADPH, nicotinamide adenine dinucleotide phosphate.

All metabolic pathways generate ATP as the primary cellular energy substrate, and nicotinamide adenine dinucleotide phosphate (NADPH) and flavin adenine dinucleotide (FADH_2_) or other biosynthetic intermediates for redox balance.^24^ These metabolic pathways differ distinctly in ATP yield, speed, and environmental adaptability, dictated by the cause of underlying immune system activation. The three main pathways for ATP production are glycolysis, oxidative phosphorylation (OXPHOS), and fatty acid oxidation (FAO) (Fig. 1a). Glycolysis rapidly converts glucose to pyruvate in the cytosol, yielding a net 2 ATP per molecule of glucose. In aerobic conditions, pyruvate is converted to acetyl-coenzyme A (acetyl-CoA) and enters mitochondria to enter the tricarboxylic acid (TCA) cycle and OXPHOS, yielding 30–36 ATP with high efficiency but slow kinetics.^23^^,^^25^ In anaerobic conditions, pyruvate is converted to lactate, which generates only 2 ATP. Mitochondrial FAO converts fatty acids into acetyl-CoA, nicotinamide adenine dinucleotide (NADH), and FADH_2_.^25^ FAO yields the highest stiochiometry of ATP, with more than 100 ATP possible from the oxidation of a single palmitate molecule.^23^ This makes mitochondrial FAO highly efficient for long-term energy supply, but only in the presence of oxygen.

Three further pathways are essential for cell function. Fatty acid synthesis is an ATP-consuming process that converts acetyl-CoA into fatty acids, needed for membrane synthesis and signalling lipids, essential for immune cell proliferation and differentiation. The pentose phosphate pathway generates ribose-5-phosphate from glucose for nucleotide synthesis and NADPH for redox balance. NADPH is essential during oxidative stress for the neutralisation of reactive oxygen species (ROS) and for maintaining reduced glutathione as a ROS scavenger.^23^^,^^26^ Finally, metabolism of amino acids such as glutamine and arginine supports functions such as the TCA cycle, cell proliferation, and the redox system.^23^

### Metabolic plasticity of innate and adaptive immunity upon activation

At steady-state, immune cell subsets favour specific metabolic pathways matching their baseline functions (Fig. 1b). For example, macrophages and T cells rely on OXPHOS and FAO when quiescent, whereas neutrophils rely on OXPHOS and glycolysis.^27^^,^^28^ To meet the rapid need for ATP after activation, innate immune cells, such as neutrophils and monocytes, switch from OXPHOS to time-saving glycolysis, fuelling cytokine production, phagocytosis, and other functions.^29^ This phenomenon is termed the ‘Warburg-like’ switch.^29^ Inflammation, whether caused by infection or by surgical injury, are at risk of hypoxia owing to high metabolic activity and vascular leakage.^29^ Therefore, anaerobic glycolysis can be favoured by innate cells, allowing for energy supply in hypoxia, but also increasing levels of lactate as a metabolite.^23^^,^^27^

The adaptive immune system, evolved to allow for anti-inflammatory counterbalance, relies on a different profile of metabolic pathways upon activation. Effector T cells engage both glycolysis and OXPHOS, and the amino acid pathway to replenish the TCA cycle.^23^^,^^27^ Memory T cells, regulatory T cells, and innate anti-inflammatory macrophages rely on OXPHOS and FAO for their long-term survival and modulating activity. Although slower, these pathways are highly efficient in ATP production and result in minimal production of inflammatory byproducts, making them ideal for quiescent, anti-inflammatory functions.^30^^,^^31^

Further adaptations become relevant under specific circumstances. Chronically inflamed tissues, for example in obesity and diabetes mellitus, can become glucose-depleted over time because of innate immune activity and constant counter-regulation through anti-inflammatory adaptive immune processes.^29^ This makes adaptive immune cells reliant on FAO and amino acid catabolism to sustain their anti-inflammatory function. During oxidative stress, immune cells increase flux through the pentose phosphate pathway to generate more NADPH.^29^ This enhanced NADPH production supports antioxidant defences and helps restore redox balance by counteracting ROS. These distinct adaptations highlight how metabolic plasticity is essential to immune function and shaped by cell subtype *and* environment.

### Control mechanisms of metabolic switching

Metabolic plasticity is regulated by interlinked immunometabolic regulators responding to specific cues, such as levels of ATP and hypoxia.^32^ The mechanistic target of rapamycin (mTOR) protein is a central master metabolic regulator of immune cell function and proliferation when ATP is available in abundance.^33^ In contrast, adenosine monophosphate-activated protein kinase (AMPK) is activated during ATP depletion, supporting catabolic pathways such as FAO and glycolysis while inhibiting mTOR activity.^34^ Both AMPK and mTOR support autophagy in states of nutrient scarcity, allowing immune cells to recycle intracellular components to provide substrate for ATP generation.^35^ Hypoxia and bacterial infection activate hypoxia-inducible factor 1-alpha (HIF-1α), driving glycolytic programming and promoting pro-inflammatory gene expression.^36^^,^^37^ Peroxisome proliferator-activated receptors (PPARs) are major regulators of lipid metabolism and modulators of anti-inflammatory responses.^38^ For example, PPARy activation reduces inflammatory cytokine release, inhibits the inflammasome, and induces an anti-inflammatory phenotype in macrophages.^39^

Crucially, metabolic byproducts not only serve as alternative energy substrates but also as immunoregulatory signals. Lactate can not only be converted back to pyruvate, yielding 12-15 ATP,^40^ but also suppresses the proliferation and cytokine production of adaptive cells,^41^^,^^42^ and pro-inflammatory signalling in macrophages.^43^^,^^44^ Ketone bodies, such as β-hydroxybutyrate and acetoacetate, are produced in low-glucose states.^45^ Compared with glycolysis, the rate-limiting enzyme in ketolysis, succinyl CoA-oxoacid transferase, is not directly regulated by hypoxia or inflammation, delivering up to 22.5 ATP per unit of β-hydroxybutyrate when oxygen delivery is compromised.^46^^,^^47^ Importantly, ketone bodies also act as signalling metabolites modulating histone acetylation and gene expression suppressing the activity of the NLPR3 (nucleotide-binding oligomerisation domain, leucine-rich repeat, and pyrin domain-containing protein 3) inflammasome.^48^

## Immune metabolic dysfunction in metabolic disease

Metabolic disease and obesity are increasing in incidence and are a major concern for public health with direct consequences for perioperative and critical care medicine.^18^ The expansion of adipose tissue in metabolic disease is a central driver of chronic low-grade inflammation. Depending on the anatomical site, adipose tissue expands by hyperplasia and hypertrophy of adipocytes with two central consequences: a vicious cycle of insulin resistance and leucocyte recruitment.^49^ Hypertrophic adipocytes become insulin resistant by overload with triglycerides and produce high levels of chemokines, resulting in recruitment of circulation-derived monocytes. Monocytes differentiate to adipose tissue-specific macrophages (ATMs), which are activated by glucose, insulin, and free fatty acids.^50^ ATMs depend on glycolysis, an effective adaptation to their hypoxic, inflamed niche,^51^ and induce further neutrophil and monocyte recruitment, and insulin resistance in adipocytes.^49^^,^^52^ Insulin-resistant adipocytes release free fatty acids in excess, further stimulating inflammatory signalling and fuelling the pro-inflammatory immunometabolic cycle.^52^

Systemically, patients show signs of chronic inflammation by monocytosis and neutrophilia combined with decreased numbers of circulating regulatory T cells.^53^, ^54^, ^55^ This is a result of haematopoietic stem cell differentiation bias towards myelopoiesis, common with increasing age and accelerated by metabolic disease.^56^^,^^57^ Cell function is also significantly impaired with heightened glycolysis and elevated ROS production in myeloid cells^58^ and leptin-induced T cell dysfunction.^59^ In an obese mouse model, CD4 T cells are hyperactivated, owing to the increased activity of the glycolysis-FAO metabolic axis, further contributing to inflammation.^60^ Clinically, these immune and metabolic perturbations are associated with an increased risk of cutaneous, urinary, respiratory, and surgical site infections.^61^, ^62^, ^63^ Crucially, obesity-induced immune changes persist even after weight loss or weight-cycling, suggesting the changes are permanent.^64^

**Fig 2:**
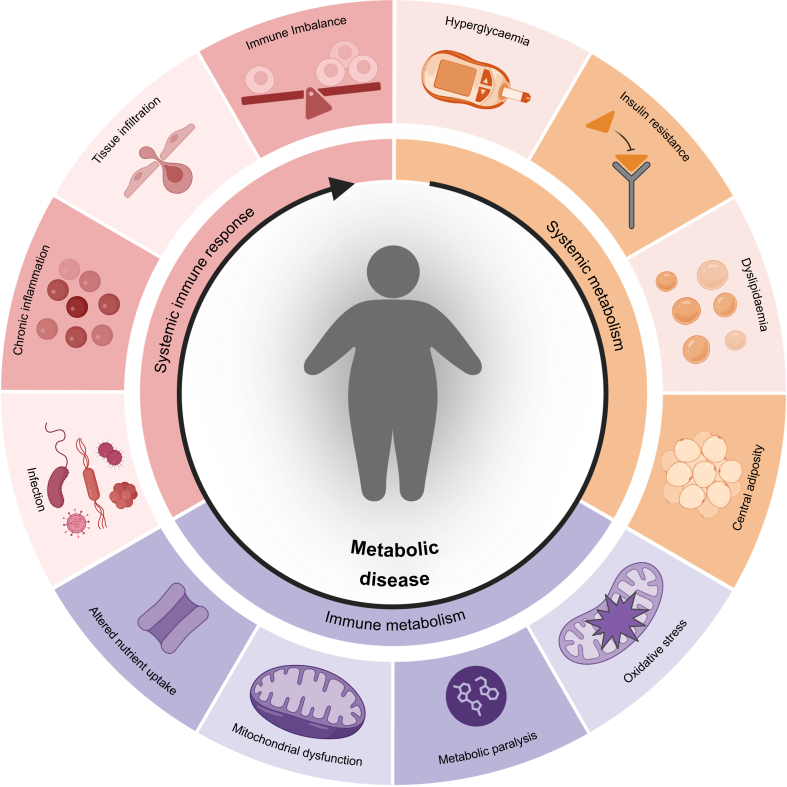
Systemic and immune metabolism alter the immune response in metabolic syndrome and ageing. Metabolic syndrome induces systemic changes, which can negatively influence immunometabolism and the systemic immune response. These shifts promote infiltration of leucocytes into tissue and immune imbalance rendering patients more susceptible to infection and chronic inflammation. Although these underlying factors are relevant to metabolic disease in general, they should also be considered in perioperative and critically ill patients as they can influence patient outcomes.

Obesity is a major risk factor for the development of type 2 diabetes mellitus (T2DM), with 90% of individuals diagnosed with T2DM also being overweight.^65^ Obesity and T2DM share many of features of immunometabolic remodelling based on insulin resistance. Diabetes mellitus accelerates the progressive age-related decline in immune function,^66^ by increased oxidative stress as a result of mitochondrial dysfunction and cellular senescence.^67^^,^^68^ Cellular senescence, a state of permanent cell cycle arrest, further promotes leucocyte-induced insulin resistance by driving chronic inflammation.^69^ Systemic hyperglycaemia is an additional important mediator of immune cell dysfunction. Hyperglycaemia induces release of ROS and neutrophil extracellular trap formation (NETosis) from neutrophils,^70^ impairs phagocytotic capacity,^71^ and delays bacterial clearance, ultimately impairing wound healing.^70^ Adaptive immunity is impaired *via* pre-activation, of naïve T cells and impaired memory T cell responses to infection,^72^^,^^73^ contributing to increased susceptibility to infections (Fig. 2).^19^^,^^20^^,^^74^

## Surgical stress-induced metabolic dysfunction and experimental challenges

Surgical injury triggers a systemic stress response, resulting in a catabolic state fuelling acute systemic inflammation and resolution.^75^ Substrate generation for ATP-synthesis is secured *via* the breakdown of carbohydrates, fats, and proteins, and reinforced by stress-induced hepatic and muscular insulin resistance.^75^ Surgical stress-induced insulin resistance leads to excessive hepatic gluconeogenesis, reduced muscular glucose uptake, and increased proteolysis, ultimately resulting in systemic hyperglycaemia.^76^ Perioperative hyperglycaemia is one of the most important independent risk factors for surgical site infection, but is also associated with other complications, such as myocardial injury.^77^, ^78^, ^79^ When systematically monitored, up to 95% of patients with diabetes mellitus and up to 60% of patients without diabetes experience perioperative hyperglycaemia.^15^ Although patients with diabetes mellitus are at higher risk of perioperative complications overall,^80^ paradoxically, when patients without diabetes experience perioperative hyperglycaemia to the same extent as those with diabetes, they are at even higher risk of postoperative complications.^15^^,^^81^ Factors contributing to the difference may include relative levels of pre-existing inflammation and insulin resistance, the magnitude of surgical injury, and the differing levels of preoperative glycaemic control. Whether these observations are connected to immunometabolism remains to be investigated, with only limited perioperative data available currently. Monocytes increase glycolysis perioperatively for rapid ATP generation to meet energy demands for effective immune defence in tissue hypoxia.^82^^,^^83^ T-lymphocytes show reduced glycolytic capacity, proliferation, and excess apoptosis after surgery, suggesting immunometabolic stunting.^84^

Although these studies are a first attempt to highlight immunometabolic adaptation during perioperative stress, they rely on study of peripheral blood mononuclear cells. This is a major limitation in perioperative translational studies, as neutrophils are the largest expanding leucocyte subset after surgery, and are not easily frozen, hindering batch analysis. Additionally, postoperative neutrophilia comprises heterogenic neutrophil subpopulations, including a significant proportion of low-density and immature neutrophils.^85^^,^^86^ There are currently no perioperative studies investigating the immunometabolic programming or dysfunction of these subpopulations; however, hypotheses could be generated from examining the findings of studies in cancer and diabetes mellitus. Neutrophils progressively lose mitochondrial capacity after release from the bone marrow and maturation.^16^ However, in chronically inflamed tumour environments, neutrophils may maintain mitochondrial capacity, eliciting the potential for higher ROS production.^16^^,^^17^ Hyperglycaemia in diabetes severely alters neutrophil functionality, impairing phagocytotic capacity, NETosis, and bacterial clearance.^71^^,^^87^ Both observations highlight the impact of the metabolic environment on neutrophil function in inflammation and metabolic disturbance that may also be relevant in the perioperative period.

Disturbed immunometabolism in the perioperative period can also negatively influence cancer survival and metastasis. Like leucocytes, cancer cells show remarkable metabolic plasticity, ultimately allowing for growth, metastasis, and immune evasion in commonly glucose-depleted local environments.^88^ Obesity, insulin resistance, and diabetes mellitus have been linked to cancer by directly influencing cancer cell growth, but also *via* suppression of anti-tumour immunity.^89^ Hyperglycaemia, and the release of insulin and catecholamines, promotes cancer cell growth, *via* the Warburg^90^ and other effects, while suppressing anti-metastatic immunity.^89^, ^90^, ^91^ Therefore, intraoperatively, we are concerned not only about surgical metastatic seeding of cancer cells, but also by the potential influence of the neuroendocrine stress response on cancer and immune cells.^91^ Whether perioperative hyperglycaemia contributes to metastatic seeding and therefore long-term cancer survival, directly or *via* impaired immune suppression, remains to be investigated. These studies are difficult to design, as they require processing of blood samples and ideally also cancerous tissue. Significant heterogeneity in cancer entities and underlying comorbidities will complicate sample size calculations and interpretation of results. Small sample size is a commonly observed limitation in experimental translational studies. Next-generation sequencing techniques will be crucial to enable data delivery at scale, albeit at significant cost.

## Immunonutrition and immunometabolic modulation

Immunonutrition aims to provide nutrients associated with immune system modulation in greater amounts than can be achieved with a standard diet or enteral feeding in the ICU.^92^ In this context, immunomodulation may either support adaptive immunity and thereby intrinsic anti-inflammatory counter-regulation, directly suppress excessive pro-inflammatory signalling, or simply act off-target by systemically altering metabolism. As many studies report associations rather than causal mechanisms, the pathways through which immunonutrition exerts its effect often remain unclear. The evidence demonstrating the utility of immunonutrition is largely derived from critical care research with limited robust data in the perioperative setting. However, studies in critical care offer insights that may be applicable in the perioperative period.

### Fasting and carbohydrate loading

**Fig 3:**
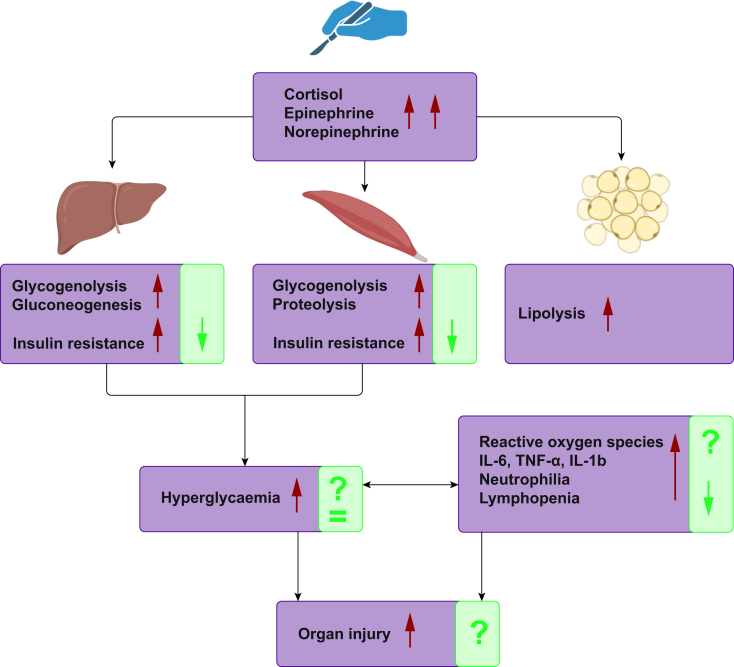
The perioperative stress response and mechanisms of carbohydrate loading. Perioperative stress results in a catabolic state with fasting and stress induced insulin resistance, ultimately leading to hyperglycaemia. Hyperglycaemia drives inflammation with increasing inflammatory cytokines, and reactive oxygen species. Findings and proposed effects of carbohydrate loading are indicated by green boxes. IL-6/1b, interleukin 6/1b; TNF, tumour necrosis factor.

Fasting has been shown to have anti-inflammatory effects *via* reduction of glycolysis and cytokine production in immune cells.^48^^,^^93^ However, the impact of fasting before or during an acute inflammatory event, such as a viral or bacterial infection, is potentially deleterious.^94^ Patients fast from solids before anaesthesia and surgery, sometimes for significantly longer than current guidelines recommend, which can contribute to fasting-induced insulin resistance and metabolic stress.^76^^,^^95^ Carbohydrate loading is not strictly considered to be immunonutrition, as carbohydrate needs can be met through diet or enteral feeding. However, the potential of immunomodulation lies in the reduction of fasting-induced insulin resistance.^96^ Carbohydrate loading is associated with a reduction in postoperative levels of inflammatory markers, neutrophilia, and increased postoperative lymphocyte counts compared with fasting patients (Fig. 3).^97^, ^98^, ^99^ Although these findings suggest an overall anti-inflammatory effect, no studies have investigated the impact on cell functionality, such as ROS production or phagocytosis. Even though patients ingest a significant amount of glucose before surgery, carbohydrate loading does not seem to induce postoperative hyperglycaemia,^99^ and is safe in patients with controlled diabetes,^100^ although studies including serial glucose measurements are currently lacking. Ultimately, whether carbohydrate loading reduces infectious complications remains unclear, with heterogeneous and underpowered study design reducing the reliability of studies.^96^

### Ketogenic diet

**Fig 4:**
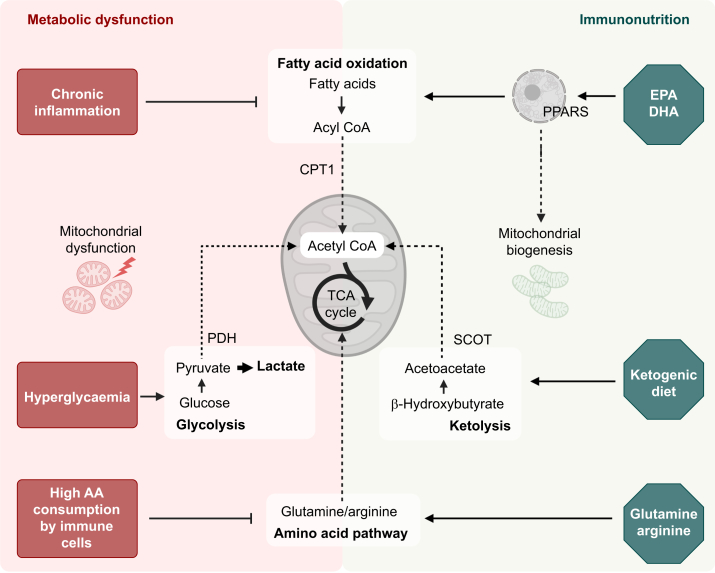
Effects of selected immunomodulatory dietary interventions. Metabolic stress in critical illness and the perioperative period includes inflammation, hyperglycaemia, and high nutrient consumption, leading to immunometabolic dysfunction (red, left panel). This impairs fatty acid oxidation (FAO), oxidative phosphorylation of glucose, and amino acid (AA) metabolism. Together with induced metabolic dysfunction, this results in reduced adenosine triphosphate (ATP) generation *via* the tricarboxylic acid (TCA) cycle and influences immune cells and their function. Targeted immunomodulatory diets (green, right panel) aim to restore mitochondrial function and immune homeostasis, by providing alternative substrates such as fish oils (eicosapentaenoic acid [EPA] and docosahexaenoic acid [DHA]), ketone bodies, or glutamine and arginine. These interventions might alleviate the immunometabolic dysfunctions underlying organ failure and impaired recovery; however, their effects depend on the clinical context, timing, dose, and route of administration. CoA, coenzyme A; CPT1, carnitine palmitoyl transferase 1; PDH, pyruvate dehydrogenase; PPAR, peroxisome proliferator-activated receptor; SCOT, succinyl-CoA:3-ketoacid CoA transferase.

Ketogenic diets are skewed towards high-fat over high-carbohydrate content, with adequate protein ratio, mimicking fasting with sufficient caloric intake.^101^ Ketolysis offers metabolic advantages in critical illness by reducing proteolysis, operating independent of insulin and providing more ATP in comparison with lipolysis (Fig. 4).^45^ Clinically, ketogenic diets are associated with fewer hypo- and hyperglycaemic events and reduced days spent on ventilation and vasopressors in intensive care.^102^ Transcriptomic analyses of T cells comparing patients with and without ketogenic diet suggest reduced T cell activation with ketosis, and reduced levels of pro-inflammatory cytokines after stimulation of whole blood *in vitro*.^102^ The immunomodulatory effects of ketogenic diet may therefore be both systemic and cell-specific. The Alternative Substrates In the Critically Ill Subject II (ASICS II) trial (ISRCTN17166453) will evaluate the effect of a 10-day ketogenic enteral feeding regimen on muscle wasting in critically ill patients by assessing the number of sit-to-stand repetitions performed in 30 s, 30 days after randomisation. The study also includes mechanistic analyses of biomarkers of catabolism and serum metabolomic assessments, allowing for granular insight into metabolomic changes with ketogenic diets. Perioperatively, ketogenic diets have been used for weight reduction before bariatric surgery with associated benefits attributed to weight loss.^103^ However, whether beneficial effects may also result from the reduction of immunodysfunction in people living with obesity remains to be investigated.

### Omega-3 fatty acid supplementation

Omega-3 fatty acids are precursors of immunomodulatory lipid mediators and activators of transcription factors out of the PPAR family.^104^ Supplementation has been associated with reduction in nosocomial infections,^104^ shortening of length of stay, and reduced risk of sepsis^105^ (Fig. 4), and is approved for use in critical care by the European Society for Clinical Nutrition and Metabolism (ESPEN).^106^ However, a clear mechanistic link is currently underexplored. The Hospital-acquired Infection Prevention with Peripheral Omegaven® (HIPPO) trial (ISRCTN11451475) is currently investigating the maximum tolerable dose of the therapeutic fish oil emulsion Omegaven® (Fresenius KABI AG, Bad Homburg, Germany) in critically ill patients not relying on parenteral nutrition. Furthermore, HIPPO will collect data on hospital-acquired infections, assess incorporation of omega-3 fatty acids in tissues, by investigating blood cells as a surrogate marker, and will explore immunomodulatory effects on leucocytes. Perioperative studies using omega-3 supplementation have differed greatly in dose, and length and form of administration. A recent systematic review claims no clear benefit of supplementation; however, heterogeneity of trial design reduces interpretation significantly.^107^

### Arginine and glutamine supplementation

Arginine and glutamine are essential amino acids that are regularly depleted in critically ill patients as a result of high consumption by immune cells and peripheral tissues (Fig. 4).^108^ Arginine regulates immune function in macrophages as a substrate for pro-inflammatory nitric oxide synthase and anti-inflammatory arginase.^109^ Furthermore, arginine is essential for proliferation, maturation, and differentiation of lymphocytes.^109^ Consequently, reduced levels of arginine result in T cell and macrophage dysfunction, impairing immune function and wound healing.^109^ Whether an increase in arginine plasma levels by parenteral nutrition benefits patients with critical illness is unclear, with results ranging from associations of increased severity in sepsis and multi-organ failure^110^ to decreased infectious complications and mortality.^109^^,^^111^ Studies showing association with benefit are often small and are confounded by use of complex immunonutritional formulations with further active nutrients, such as omega-3 fatty acids, glutamine, or nucleotides. A more recent study suggested that an arginine-rich formula is effective in reducing ICU lengths of stay.^112^ Providing the arginine precursor citrulline was associated with improved biochemical measures, reduced duration of invasive ventilation, lower Sequential Organ Failure Assessment (SOFA) score, and increased days alive at 28 days.^113^ Concerns about arginine-induced increase of nitric oxide, and thereby excessive vasodilation and haemodynamic instability, have been refuted.^109^ However, a transient increase in intra-abdominal pressure 24 h after arginine infusion, attributable to regional fluid shifts, could reduce mucosal perfusion.^114^ The Randomised Trial of Immunonutrition with L-citrulline in Patients Hospitalised in Intensive Care for Sepsis or Septic Shock (ICITRU) trial (NCT04513288) is investigating the effects of enteral supplementation with the arginine precursor L-citrulline over 5 days in patients with sepsis, with SOFA score as primary outcome. Further investigations include measurement of immunological parameters, such as in-depth leucocyte characterisation with human leucocyte antigen–DR isotype (HLA-DR) expression and mitochondrial functionality test, systemic cytokine levels, and enumeration of specific leucocyte subpopulations, such as myeloid-derived suppressor cells and lymphocytes. In summary, arginine supplementation at levels greater than in standard enteral or parenteral nutrition is currently not recommended owing to lack of clear and consistent improvements in patient outcomes.^115^

Glutamine is also a key immunomodulatory amino acid, supporting immune cell energy metabolism, proliferation, and function. Examples include T cells requiring glutamine for proliferation, B cells for the differentiation into antibody secreting plasma cells, and macrophages for major histocompatibility complex class II (MHC-II) expression with subsequent antigen presentation and effective phagocytosis.^116^ The Glutamine and Antioxidant Supplementation in Critically Ill Patients (REDOXS) trial reported an association of early glutamine supplementation with higher mortality,^117^ in contrast to previous studies, indicating reduced mortality and complications.^118^ The authors hypothesised that high glutamate dose, patient selection, and early initiation of supplementation may have contributed to these results.^117^ A re-analysis of the REDOXS trial revealed higher urea-to-creatinine ratio in patients receiving glutamine supplementation, potentially mediating the increased risk of death.^119^ In this instance, glutamine was not utilised for energy and ultimately converted to urea, increasing risk for hyperammonaemia and mortality.^119^ This highlights that benefits may not be observed owing to downstream disturbed physiological processes during illness. Considering the current inconsistent findings, guidelines do not recommend immunonutrition for all critically ill patients, but for specific conditions, such as glutamine supplementation in burn and trauma patients.^106^

Studies in the perioperative period have administered amino acid formulations differing widely in dose, length of intervention, and formulation. Surgical patients receiving arginine and glutamine for 7 days before enterocutaneous fistula surgery had better postoperative recovery, indicated by fewer infectious complications, less fistula recurrences, and lower levels of interleukin-6 (IL-6) and C-reactive protein (CRP).^120^ In patients undergoing colorectal cancer surgery, perioperative arginine supplementation preserved natural killer cell cytotoxicity but not interferon-gamma (IFN-γ) secretion.^121^ A meta-analysis on immune function and postoperative complications in patients with colorectal cancer indicated that glutamine supplementation may improve surgical site infection, anastomotic leaks, and length of hospital stay.^122^ This was mechanistically associated with improved immunoglobulin response, T cell function, and reduced CD4+/CD8+ ratio.^122^ Many immunonutritional formulations are heterogeneous, for example combining omega-3 fatty acids with arginine or antioxidants. Recent meta-analyses of mixed formulations applied in surgical patients have shown reductions in urinary tract and surgical site infections, and anastomotic leaks and length of hospital stay.^123^^,^^124^ However, the 2025 updated ESPEN guidelines on nutrition in surgery state that arginine-containing immunonutrition may be considered in malnourished patients undergoing major gastrointestinal cancer surgery only, and that glutamine supplementation is not recommended routinely.^100^ The recommendation only received a grade B, as many studies were performed before the implementation of enhanced recovery after surgery protocols. The Immunonutrition in ERAS protocols in Gynaecologic Oncology (NUTRIGO) trial (NCT06103526) is currently investigating immunonutrition supplements for 3 days pre- and post-surgery and its effect on perioperative infections and length of hospital stay, but is not providing a breakdown of the formulation.

## Further pharmacological interventions with the potential to modulate immunometabolism

### Anaesthetic agents

Whether the mode of anaesthesia influences outcome overall or cancer survival remains under discussion. *In vitro* data have shown that isoflurane promotes cancer cell growth in comparison with propofol,^125^ and also induces immune cell cytotoxicity.^126^ However, these effects have not been confirmed in clinical trials and remain under investigation.^127^^,^^128^ However, mode of anaesthesia may also be of relevance to other patient groups at risk, for example those with insulin resistance. Compared with total i.v. anaesthesia with propofol, maintenance of anaesthesia with desflurane and sevoflurane in patients with diabetes mellitus has been associated with higher blood glucose and cortisol levels, and lower insulin levels by the end of surgery.^129^ Sevoflurane has also been associated with higher levels of inflammatory markers, such as IL-6, in comparison with propofol.^130^ Patients without diabetes mellitus randomised to sevoflurane over propofol maintenance had higher circulating levels of cortisol and glucose throughout surgery.^131^ These observations are in line with sevoflurane-induced insulin resistance in studies of large and small mammals fuelling hyperglycaemia.^132^^,^^133^ Concerning immune cells, sevoflurane disrupts the mitochondrial membrane potential and may thereby induce apoptosis and oxidative stress in leucocytes.^134^^,^^135^ Further insight into the impact of mode of anaesthesia overall and on glucose metabolism in particular is anticipated from the Volatile *vs* Total intravenous Anaesthesia for major non-cardiac surgery (VITAL)^136^ and GlucoVITAL^137^ trials, both randomising patients to inhalation or propofol-based maintenance of anaesthesia. The primary outcome for VITAL is days alive and at home within 30 days after surgery, survival, and quality of life.^136^ The primary outcome for GlucoVITAL is blood glucose at the end of surgery relative to the start of surgery.^137^ In addition, patients enrolled in GlucoVITAL wear continuous glucose monitoring.^138^ The trial therefore addresses several questions: whether the mode of anaesthesia plays a role in metabolic dysfunction and whether glycaemic variability is associated with perioperative complications.

### Metformin

Metformin therapy before sepsis has been associated with improved survival without an increase in lactate.^139^^,^^140^ Perioperatively, metformin therapy may reduce mortality^141^ and complications.^142^ The mechanisms behind these observations remain unclear but may be both metabolic and immunomodulatory. One central mechanism of action of metformin is inhibition of the mitochondrial respiratory chain, leading to intracellular AMP accumulation and AMPK induction.^143^ This results in reduced gluconeogenesis in hepatocytes and a raft of anti-inflammatory effects observed in adipose tissue, endothelial cells, and leucocytes.^143^ Among the main explored mechanisms are reduction in nuclear factor kappa-light-chain-enhancer of activated B cells (NF-kB) activity and thereby reduced production of pro-inflammatory cytokines, and reduced NLPR3 inflammasome activation.^143^ Not all anti-inflammatory mechanisms of metformin are direct consequence of AMPK modulation,^143^ but it is a central example of the tight connection between metabolism and inflammation. The Randomized Clinical Trial of the Safety and FeasibiLity of Metformin as a Treatment for sepsis-associated acute kidney injury (LiMiT AKI) trial (NCT05900284) hypothesises that metformin-mediated activation of AMPK protects against sepsis-associated acute kidney injury and death by limiting mitochondrial injury.^144^ Besides the safety and feasibility data collected as primary outcomes, the trial will also look at mitochondrial function and bioenergetics of platelets. Although the researchers suggest that the underlying mechanism might include modulation of the inflammatory response, there is currently no planned analysis in other cell types of the immune system.

Two further studies are evaluating the effect of metformin (Evaluation of the Effect of Metformin on the Clinical Outcome of ICU Patients with Sepsis [NCT05979038] and Metformin and Lactoferrin in Sepsis in ICU [NCT06181422]). Neither explores mechanisms. One study (Metformin Use in Patients Undergoing Total Joint Replacement Surgery [NCT06280274]) is currently randomising patients with and without diabetes having total joint replacement to 14 days of preoperative metformin and assessing surgical site infection. These studies highlight that long-established medications may hold further therapeutic potential, when investigated within a new context.

### Dexamethasone

Dexamethasone is commonly administered perioperatively as a prophylaxis for nausea and vomiting. It transiently increases blood glucose; however, this seems to be of questionable clinical metabolic relevance with a mean difference of 1 mM in blood glucose, 4 h after administration.^145^ Hyperglycaemic events, with blood glucose >10 mM, are more frequent in patients receiving dexamethasone, but are still uncommon, with an event rate of 2.7% after dexamethasone compared with 1.2% in control patients.^145^ Crucially, patients with and without diabetes experience an increase in blood glucose after dexamethasone administration.^145^ Dexamethasone shapes immunometabolism by inhibiting HIF-1α-induced glycolysis, rendering macrophages less pro-inflammatory.^146^ Larger doses of dexamethasone before cardiopulmonary bypass reduced the pro-inflammatory response.^147^ Interestingly, the dexamethasone-induced suppression of pro-inflammatory cytokine release seems to be uncoupled from antimicrobial defence.^148^ This observation is consistent with the Perioperative Administration of Dexamethasone and Infection (PADDI) trial, which randomised patients to dexamethasone or placebo, and which showed no increased risk of postoperative surgical side infection in all patients or in patients with diabetes mellitus.^149^ The median (interquartile range) difference in blood glucose level between the baseline and the maximum value up to postoperative day 2 was 3.6 (2.5–4.9) mM with dexamethasone and 2.4 (1.4–3.6) mM in dexamethasone and placebo patients, respectively.

## Conclusions

Immunometabolic plasticity is central to immune cell functionality and ultimately inflammatory response and resolution. Upon activation, immune cells undergo rapid metabolic reorganisation to optimise energy needs while efficiently orchestrating a balanced immune response. Both critical illness and the perioperative period serve as models of translation for many questions of acute inflammation but are currently underutilised. This is partly a reflection of the difficulties faced in conducting these studies. Patients in both fields present with high heterogeneity and often with chronic disturbed immunometabolism. Inflammation and immunometabolism are highly dynamic throughout different stages of surgery and critical illness, rendering single measurements misleading. Sampling of tissues is constrained, with assumptions made from analyses performed on circulating leucocytes and plasma. However, as highlighted, immunometabolism is highly dependent on local tissue cues resulting in significant differences in metabolic programming in circulating and tissue-resident cells. Studies using novel techniques have overcome some of these challenges, allowing for serial measurements and at-scale sequencing. Advancing our understanding of immunometabolic regulation in the perioperative period and critical illness using cell-specific approaches will further our knowledge of acute inflammatory challenges, characterise chronic underlying inflammation present in a significant number of our patients, and ultimately, identify when and for whom immunometabolic interventions may meaningfully improve outcomes.

## Authors’ contributions

Writing of manuscript: all authors

Read and approved final version: all authors

## Funding

British Journal of Anaesthesia/Royal College of Anaesthetists (BJA Centenary grant 2024-27 to HJ).

## Declarations of interest

IN is part of the study team for HIPPO and ASICS II. HJ is part of the study team for GlucoVITAL. The other authors declare that they have no conflicts of interest.
